# Application of 3D‐Slicer combined with 3D printing technology in training neurosurgery resident physicians' imaging spatial cognition and clinical reasoning: A self‐controlled before‐ and after‐study

**DOI:** 10.1002/ase.70321

**Published:** 2026-08-13

**Authors:** Li Xueyu, Fan Yuanxian, Chen Jiaxing, Li Chuanyu, Chen Yuanbiao, Huang Haineng, Huang Huadong, Luo Kunxiang, Wei Shiyin, Tan Rong

**Affiliations:** ^1^ Affiliated Hospital of Youjiang Medical University for Nationalities Baise Guangxi Zhuang Autonomous Region China; ^2^ Key Laboratory of Biomedical Material Research of Guangxi (Cultivation) Baise Guangxi Zhuang Autonomous Region China

**Keywords:** 3D printing, 3D Slicer, clinical reasoning, medical education, resident education

## Abstract

Neurosurgical residency training requires advanced spatial cognition and 3D anatomical understanding, which traditional methods often fail to adequately develop. This study evaluated a novel educational model integrating 3D Slicer and 3D printing technology to enhance imaging spatial cognition and clinical reasoning among neurosurgery residents. In a self‐controlled before‐ and after‐trial, 25 neurosurgery residents completed a 12‐week training program involving 3D Slicer reconstruction and 3D printing of clinical cases. Core competencies were assessed at baseline (T0) and post‐intervention (T1) through theoretical tests, spatial anatomical recognition tasks, and clinical case analysis. Subjective feedback was collected via a structured survey. Participants showed significant improvements in all assessment domains (*p* < 0.001). Spatial anatomical recognition scores increased by 16.96 points (from 67.12 to 84.08), clinical case analysis by 13.10 points (from 69.94 to 83.04), and theoretical knowledge by 12.32 points (from 72.32 to 84.64). Subjective evaluations indicated that 100% of residents reported moderate or significant improvement in 3D anatomical comprehension, spatial visualization, preoperative planning confidence, and proactive problem‐solving. The integrated 3D Slicer and 3D printing training model effectively enhances spatial cognition, clinical reasoning, and active learning in neurosurgical education. It offers a scalable, clinically relevant approach to bridging the gap between 2D imaging interpretation and 3D surgical planning, supporting the transition from training to independent practice.

## INTRODUCTION

Residency standardized training represents a critical initiative in healthcare reform and medical education advancement, serving as an essential pathway for medical graduates to develop into competent clinical practitioners.^1^, ^2^ Neurosurgical residents enter specialized training with foundational knowledge of neurological disorders acquired during undergraduate education; however, they encounter a discipline requiring exceptional spatial cognition and three‐dimensional comprehension of complex intracranial anatomy.^3^, ^4^ With the rapid evolution of imaging technologies, surgical procedures have grown increasingly complex, while training duration remains relatively constrained.^1^, ^5^ Traditional apprenticeship‐based teaching methodologies prove inadequate for meeting the demands of standardized neurosurgical training, particularly in cultivating the spatial reasoning, precision, and visualization skills essential for contemporary practice.^1^, ^3^ Furthermore, these conventional approaches demonstrate limitations in stimulating learning engagement and motivation among trainees. These challenges are particularly pronounced in underdeveloped regions, such as Western China. Due to economic constraints and a significant ‘brain drain’ of highly qualified medical professionals to major metropolitan centers, grassroots hospitals bear the immense burden of cultivating competent neurosurgeons with severely limited resources. Consequently, there is an urgent need for an effective, low‐cost educational model that can democratize high‐level neurosurgical training for resource‐limited settings.^6^

The integration of anatomical imaging with innovative technologies offers a promising solution to these educational challenges. For instance, quantitative anatomical imaging has demonstrated its utility in elucidating structural relationships and guiding clinical decisions in various medical contexts.^7^ In neurosurgical education, tools like 3D Slicer enable the transformation of standard DICOM data into detailed virtual models, while 3D printing produces tactile, patient‐specific phantoms that enhance hands‐on learning and spatial recognition. Such methodologies align with contemporary educational paradigms that emphasize experiential and multimodal learning, as seen in studies where anatomical imaging improves diagnostic accuracy and preoperative planning.^7^, ^8^, ^9^

Despite these advancements, the systematic incorporation of combined 3D Slicer reconstruction and 3D‐printed models into neurosurgical residency programs remains underexplored, particularly in standardized training settings.^7^, ^9^ Current literature lacks comprehensive evaluations of how this integrated approach specifically enhances imaging spatial cognition and clinical thinking abilities among residents during their critical transition to independent practice.^7^, ^8^ This study aims to bridge this gap by investigating the efficacy of a novel training model that leverages 3D Slicer and 3D printing to foster these competencies in neurosurgery residents, with the hypothesis that this approach will significantly improve anatomical understanding, spatial reasoning, and surgical confidence.

## METHODS

### Study participants

A total of 25 neurosurgery resident physicians were enrolled in this study between January 2023 and July 2025.

*Inclusion Criteria*:

Completion of 24 months of basic neurosurgery rotations.

No prior exposure to 3D printing or 3D Slicer software training.

Ability to fully participate in the 3‐month training program and complete both pre‐ and post‐training assessments.

Willingness to provide informed consent.

*Exclusion Criteria*:

Absence exceeding 7 days during the training period.

Previous professional neurosurgery work experience.

All participants were male, reflecting the gender composition typical in this surgical subspecialty.

### Study design

A self‐controlled before‐ and after‐study design was employed to evaluate the effect of a structured 3‐month training intervention on participants' imaging spatial cognition and clinical reasoning abilities.

#### Training intervention

The 12‐week program combined theory, software training, and hands‐on practice. To ensure training did not overwhelm the residents' routine clinical ward duties, the curriculum mandated at least 1 h of dedicated, protected teaching time per week. The program was structured as follows:

*Weeks 1–2: Theoretical Foundation and Software Introduction*

To ensure reproducibility and provide a clear methodological framework for the trainees, a standardized 6‐step workflow for 3D Slicer segmentation and reconstruction was established (Table 1). This step‐by‐step protocol guided residents from raw data processing to the final extraction of printable 3D models. Covered 3D printing applications in neurosurgery and 3D‐Slicer operations (DICOM import, segmentation, volume rendering). Introduced threshold‐based and region‐growing segmentation.

**TABLE 1:** Standardized 3D‐Slicer workflow for neurosurgical case reconstruction.

Step	Phase	3D‐Slicer module/tool	Specific action and objective
1	Data import	DICOM module	Import patient‐specific thin‐slice CT, CTA, or MRI DICOM datasets. Verify spatial resolution and image orientation
2	Region of interest (ROI) definition	Crop volume module	Define a bounding box around the target surgical area (e.g., tumor, aneurysm, and planned skull window) to reduce computational load and isolate relevant anatomy
3	Primary segmentation	Segment Editor: Thresholding/Grow from seeds	Apply intensity‐based Thresholding for high‐contrast structures (e.g., skull, main arteries). Utilize Grow from Seeds (region‐growing) for complex soft‐tissue lesions like tumors or hematomas
4	Manual refinement	Segment Editor: Paint/Erase smoothing	Manually slice‐by‐slice edit boundaries to separate adjacent structures (e.g., detaching tumor margins from the brainstem or defining the aneurysm neck). Apply Smoothing (Gaussian or Median) to eliminate artifacts and create natural anatomical surfaces
5	3D visualization	Data/Volume rendering	Generate 3D closed surface representations from the 2D segments. Assign distinct colors and opacities to different structures (e.g., red for arteries, yellow for tumors, bone‐color for skull) to conceptualize the spatial relationships
6	Model export	Export to files	Export the finalized, separated 3D models as Standard Tessellation Language (.STL) or Wavefront (.OBJ) files, ensuring they are ready for slicing software and 3D printing

*Weeks 3–10: Core Skill Development and Case Reconstruction*

Trainees reconstructed five representative clinical cases (Sellar Tumor, MCA Aneurysm, Basal Ganglia ICH, Meningioma, and Vestibular Schwannoma) using 3D Slicer, focusing on lesions, vessels, and adjacent structures.

*Weeks 11–12: 3D Model Printing and Case Seminar*

Selected models were printed at a 1:1 scale. To ensure affordability in our resource‐limited setting, accessible desktop 3D printers were utilized—initially, the ANYCUBIC Vyper 3D for single‐color prints, later upgraded to the Bambu Lab P1Sc for dual‐color capabilities. The primary material used was rigid polylactic acid (PLA). While flexible materials like TPU were tested to simulate soft tissue, high printing failure rates precluded their routine use. Consequently, in this context, ‘surgical simulation’ primarily referred to cognitive and spatial planning rather than physical, hands‐on tissue dissection (such as actual aneurysm clipping). Because our cohort mainly consisted of residents from grassroots hospitals in Western China—where establishing a solid microneurosurgical foundation is the priority—the simulation focused heavily on approach trajectory and craniotomy placement. The vast majority of participants engaged in visual and spatial simulation by concurrently manipulating the 3D‐Slicer digital reconstructions, handling the 3D‐printed physical models, and analyzing corresponding intraoperative surgical videos. While a minority of advanced trainees had the opportunity to participate as first assistants in the actual surgeries, the core objective for all trainees was to master the cognitive transition from 2D imaging to 3D surgical perspectives within a structured, instructor‐led seminar.

#### Outcome measures

Assessments were conducted at baseline (T0) and immediately after training (T1) using the following tools:

##### Theoretical knowledge test (100 points)

This assessment consisted of a 50‐question multiple choice questionnaire (MCQ), with each question worth 2 points. Crucially, the exam evaluated comprehensive neurosurgical anatomy, basic pathology, and spatial diagnostics, rather than being strictly limited to the topics of the 5 specific cases that were 3D‐printed. The primary goal of this comprehensive evaluation was to assess the residents' foundational grasp of overall neuroanatomy—particularly structural adjacency and neurovascular relationships. We designed the test this way because a robust, general understanding of microneuroanatomy is a strict prerequisite for mastering the principles of safe craniotomies and fundamental surgical approaches, which is a critical necessity for trainees preparing for independent practice in resource‐limited settings.

##### Spatial anatomical recognition task (100 points)

The assessment of imaging spatial cognition utilized a standardized scoring system (Table 2). Five representative neurosurgical pathologies were selected, and for each case, trainees were required to identify five critical neurovascular or bony landmarks. To ensure objective quantification, a partial‐credit scoring method (0/2/4 points) was implemented, which has been validated in previous anatomic imaging studies. Clinical reasoning was further evaluated through a structured case analysis covering imaging interpretation, diagnostic logic, and treatment planning.

**TABLE 2:** Imaging anatomic spatial identification test score sheet.

No.	Pathology and imaging modality	3D‐printed and reconstructed components (simulation focus)	Key anatomical structures for identification	Points (per structure)
Case 1	Sellar Tumor (CTA‐MRI Fusion)	**Printed:** Sella turcica/sphenoid bone, Tumor mass, Bilateral ICAs. **Slicer:** Optic pathways	(1) Pituitary gland. (2) Optic chiasm. (3) Cavernous sinus. (4) Supraclinoid ICA. (5) Third ventricle	0/2/4
Case 2	MCA Aneurysm (CTA)	**Printed:** Pterional skull window, MCA tree, Aneurysm dome and neck **Slicer:** Parent artery trajectory	(1) M1 segment of MCA. (2) Carotid siphon. (3) ACom. (4) Lenticulostriate arteries. (5) Sylvian fissure	0/2/4
Case 3	Basal Ganglia ICH (CTA)	**Printed:** Target skull window, Hematoma volume **Slicer:** Corticospinal tract projection, Ventricular system	(1) Lentiform nucleus (2) Posterior limb of internal capsule (3) Thalamus (4) M2 segment of MCA (5) Anterior choroidal artery	0/2/4
Case 4	Meningioma (CT‐MRI Fusion)	**Printed:** Skull window, Tumor mass, Sagittal/Transverse sinus segments **Slicer:** Surrounding venous network	(1) Superior sagittal sinus. (2) Transverse sinus (3) Torcular herophili. (4) Sigmoid sinus. (5) Superior petrosal sinus	0/2/4
Case 5	Vestibular Schwannoma (CT‐MRI Fusion)	**Printed:** Petrous bone, Occipital bone (with inion), Tumor mass, Venous sinuses **Slicer:** CPA cistern, Basilar artery, Nerve bundles	(1) Transverse and sigmoid sinuses. (2) Facial‐vestibulocochlear nerve complex (CN VII/VIII). (3) External occipital protuberance. (4) Basilar artery. (5) Cerebellopontine angle cistern	0/2/4

##### Clinical case analysis (100 points)

Diagnostic and surgical plans for a novel case, evaluated by blinded experts (30% imaging, 30% diagnosis, 40% treatment).

##### Subjective satisfaction survey

A post‐training survey comprising nine items evaluated perceived enhancements in learning interest, anatomical comprehension, spatial visualization, and preoperative planning confidence on a 4‐point Likert scale (significantly improved, moderately improved, neutral, not improved).

### Statistical analysis

Statistical analyses were conducted using SPSS version 22.0. Continuous variables are expressed as mean ± standard deviation. Normality of data distribution was evaluated with the Shapiro–Wilk test due to the limited sample size (*n* = 25). For normally distributed data, paired *t*‐tests were employed, whereas the Wilcoxon signed‐rank test was utilized for nonnormally distributed data. Statistical significance was defined as *p* < 0.05.

This study was deemed exempt from formal Institutional Review Board (IRB) approval because it solely evaluated an educational curriculum enhancement. The creation of the 3D models relied entirely on fully anonymized, retrospective imaging data. The study involved no experimental clinical interventions, did not alter standard patient care, and posed no risk to participants (Figure 1).

**FIGURE 1:**
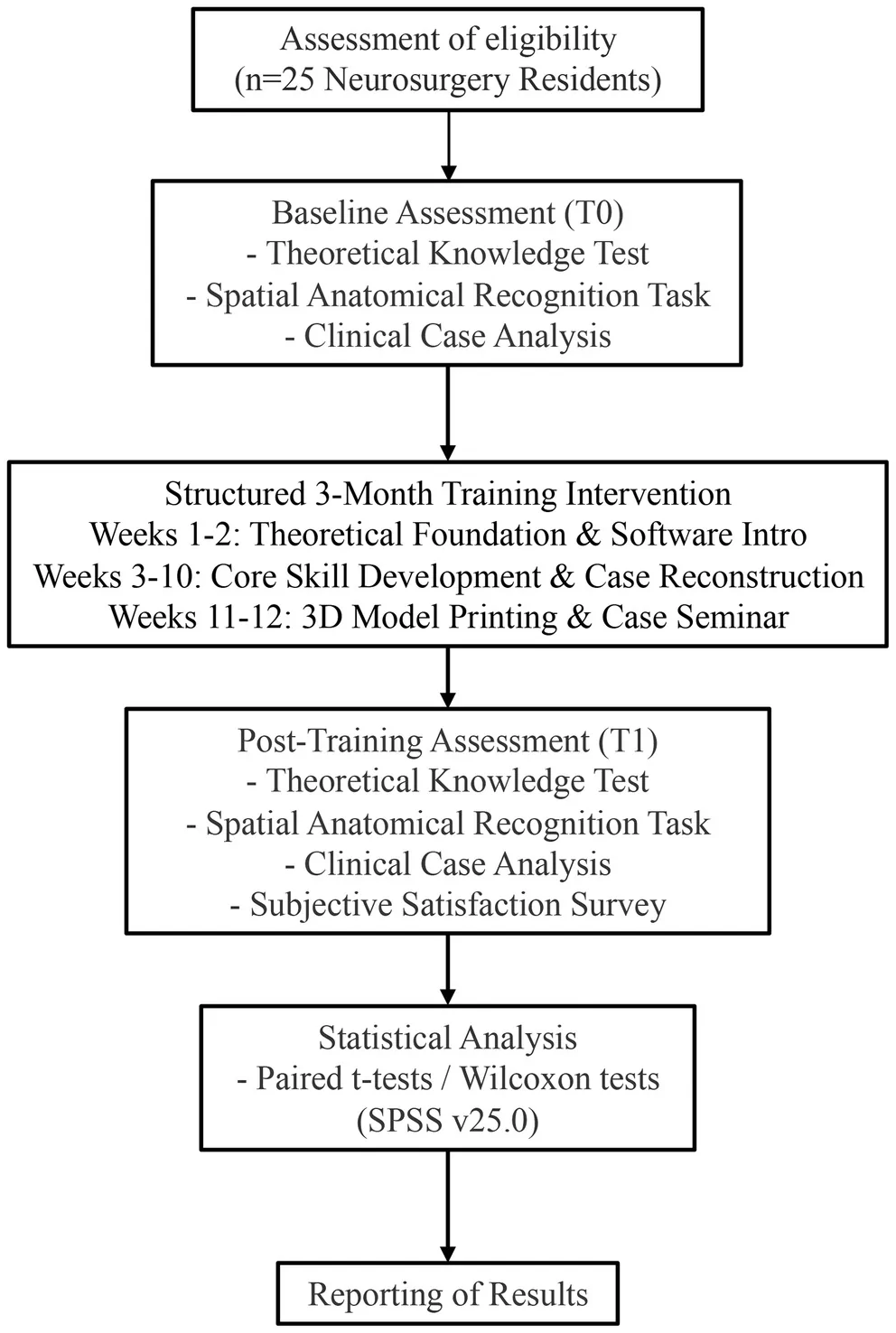
Study design and participant flow.

## RESULTS

### Study design and participant characteristics

This study adopted a self‐controlled before‐ and after‐design to evaluate the effectiveness of the training program on the core competencies of residency training residents. Two assessment time points were set: before training initiation (T0) and after training completion (T1), with a total training cycle of 12 weeks.

A total of 25 residency training residents participated in this study, among whom 16 were professional master's degree postgraduates. All participants were male. At the time of training, their ages ranged from 23 to 27 years, with a mean age of 24.88 years. Throughout the study, all 25 residents completed the full training course and attended the assessments at both T0 and T1, with no participant dropout reported.

### Descriptive statistics

#### Normality test results

Before conducting inferential statistics, the Shapiro–Wilk test was used to assess the normality of scores for the three core competence indicators (theoretical assessment, spatial anatomical recognition, clinical case analysis) at T0 and T1.

The results showed that all indicator scores conformed to a normal distribution at both time points (all *p* > 0.05), as detailed in Table 3. Therefore, the paired samples *t*‐test was selected for subsequent comparison of scores before and after training (Table 3).

**TABLE 3:** Shapiro–Wilk normality test results of core competence indicator scores.

Assessment indicator	Time point	Shapiro–Wilk statistic	*p*‐value	Normality conformity
Theoretical knowledge	T0	0.986	0.975	Yes
	T1	0.942	0.162	Yes
Spatial anatomical recognition	T0	0.974	0.743	Yes
	T1	0.939	0.141	Yes
Clinical case analysis	T0	0.992	0.999	Yes
	T1	0.964	0.501	Yes

#### Descriptive statistics

Descriptive statistics for the three core competence indicators (presented as $\bar{x}\pm S$) at T0 and T1 are given in Table 4.

**TABLE 4:** Descriptive statistics and pre‐ post‐training paired *t*‐test results of core competence indicator scores (*n* = 25).

Assessment indicator	Time point	Score ($\bar{x}\pm S$)	MD (95% CI)	*t*‐value	*p*‐value
Theoretical knowledge	T0	72.32 ± 4.66	12.32 (9.65–14.99)	−9.27	<0.001
	T1	84.64 ± 4.73			
Spatial anatomical recognition	T0	67.12 ± 4.2	16.96 (14.57–19.35)	−14.25	<0.001
	T1	84.08 ± 4.22			
Clinical case analysis	T0	69.94 ± 3.97	13.1 (10.85–15.35)	−11.70	<0.001
	T1	83.04 ± 3.95			

After training (T1): A consistent upward trend was observed in all indicators, with significant score improvements compared with T0.

#### Inferential statistical analysis results (paired samples *t*‐test)

Since all indicator scores conformed to a normal distribution, the paired samples *t*‐test was used to compare the differences between T0 and T1. The results confirmed that all three indicators showed statistically significant improvements after training (all *p* < 0.001) (Table 4).

#### Visualization of training effects

Three figures were generated to intuitively present the training effects, and their results were consistent with the statistical findings above (Figures 2 and 3).

**FIGURE 2:**
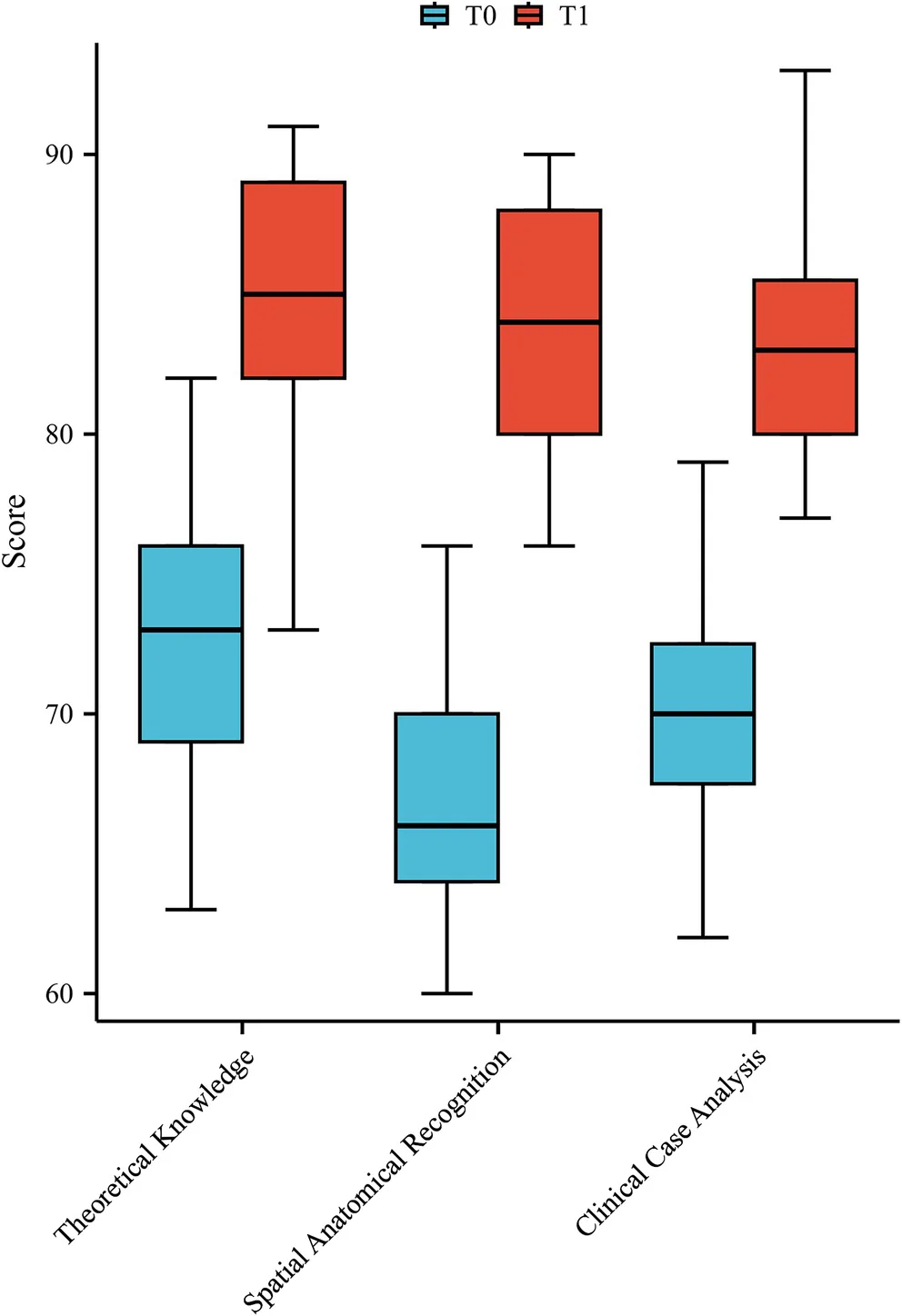
(Box Plot): Displays the distribution of scores for the three indicators at T0 and T1. The boxes (representing interquartile ranges) and medians of T1 were significantly higher than those of T0 for all indicators. No overlap of interquartile ranges was observed in spatial anatomical recognition, further confirming the magnitude of its improvement.

**FIGURE 3:**
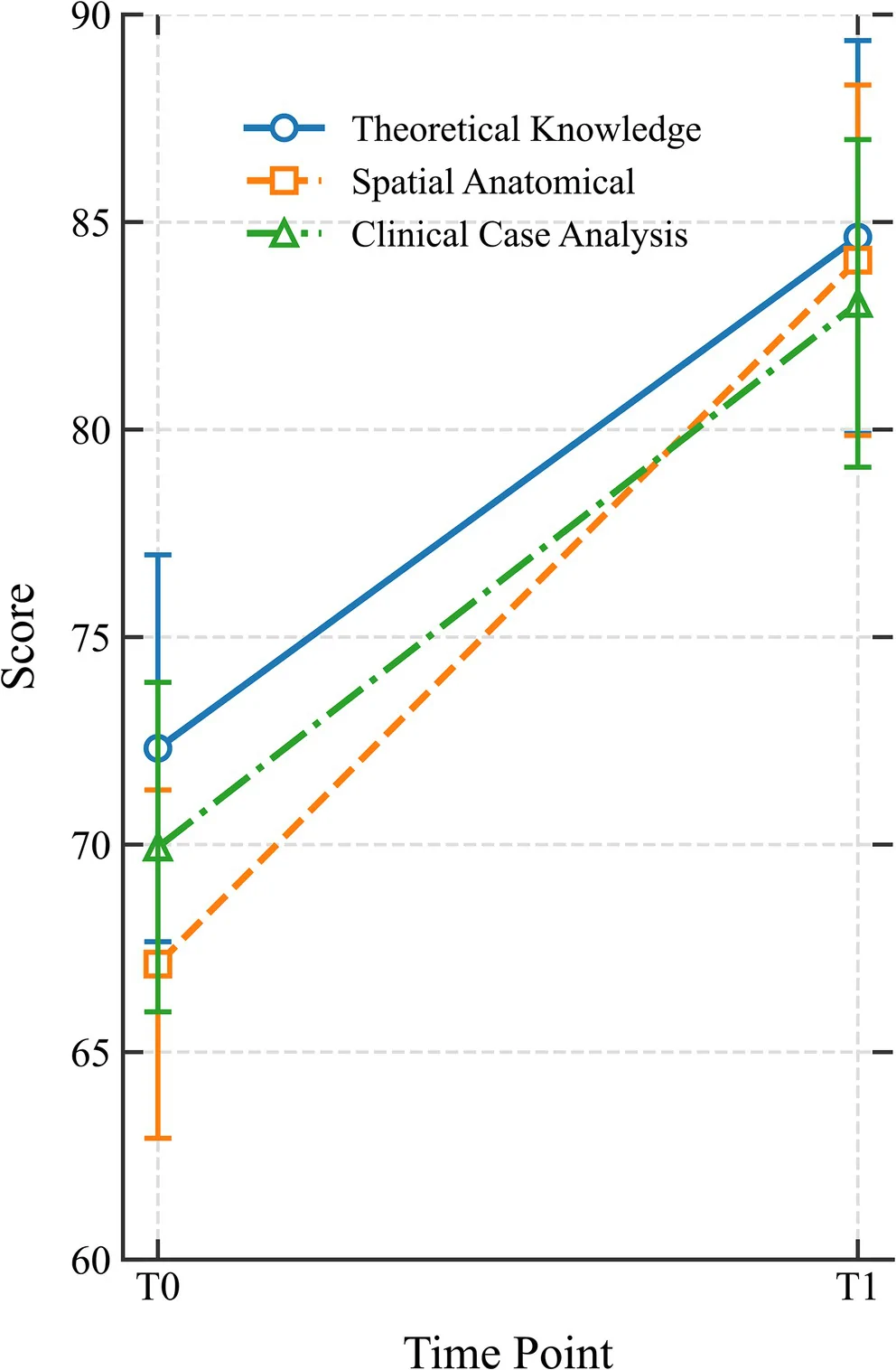
(Line Graph with Error Bars): Illustrates the trend of mean scores ($\bar{x}\pm S$) before and after training. All three lines showed a sharp upward shift from T0 to T1, with spatial anatomical recognition having the steepest slope—reflecting its most prominent improvement.

#### Qualitative evaluation (subjective feedback)

All 25 distributed questionnaires were returned (100% response rate). As given in Table 5, participants reported strong positive outcomes: 100% of respondents reported either “Significantly improved” or “Moderate improved” in all evaluated dimensions. No neutral feedback or reports of “Not improved” were recorded, indicating the model's consistent positive impact.

**TABLE 5:** Evaluation results of the neurosurgery teaching model (*n* = 25).

Survey item	Significantly improved *n* (%)	Moderately improved *n* (%)
Stimulated interest in learning neurosurgery	19 (76%)	6 (24%)
Enhanced understanding of 3D intracranial anatomy	23 (92%)	2 (8%)
Improved ability of 2D‐to‐3D spatial conceptualization from images	19 (76%)	6 (24%)
Strengthened grasp of lesion‐neurovascular spatial relationships	20 (80%)	5 (20%)
Elevated ability to interpret CTA/MRA/DSA images	19 (76%)	6 (24%)
Boosted confidence in formulating individualized surgical plans	22 (88%)	3 (12%)
Improved ability to communicate disease conditions with patients and their families	20 (80%)	5 (20%)
Promoted the ability of proactive inquiry and problem‐solving	17 (68%)	8 (32%)
Overall satisfaction with this teaching model	21 (84%)	4 (16%)

*Note*: The table reports a post‐training survey comprising 9 items evaluating perceived enhancements in learning interest, 3D intracranial anatomical comprehension, 2D‐to‐3D spatial conceptualization, lesion‐neurovascular spatial relationships, interpretation of CTA/MRA/DSA images, confidence in formulating individualized surgical plans, communication with patients and their families, proactive inquiry and problem‐solving ability, and overall satisfaction with the teaching model. All items were rated on a 4‐point Likert scale (significantly improved, moderately improved, neutral, not improved). Results showed that 100% of respondents reported either “Significantly improved” or “Moderately improved” across all dimensions, with no neutral or “not improved” responses recorded. The “Neutral” and “Not improved” columns are omitted as all values in these two columns are 0.

#### Summary of training efficacy

Collectively, the normality test, descriptive statistics, and inferential analysis (paired samples *t*‐test), combined with visualizations, demonstrate that the training program significantly enhances the core competencies of neurosurgery residents (all *p* < 0.001). Among the three indicators, spatial anatomical recognition showed the greatest improvement, followed by Clinical Case Analysis and Theoretical Knowledge—indicating that this training method is particularly effective in improving spatial cognition, a key challenge in traditional neurosurgery resident training.

The mean scores and standard deviations for each category before and after training are given in Table 4. The mean scores after training were higher than those before training for all categories.

## DISCUSSION

In the era of microneurosurgery, where surgical expertise serves as the cornerstone of advancing the discipline, standardized training for neurosurgery residents faces a core challenge: how to enable trainees to rapidly and systematically master the spatial adjacency of neuroanatomical structures and the anatomical basis of lesions.^10^, ^11^ This challenge is further compounded by the absence of mandatory requirements for practicing physician qualifications in residency admission criteria. This absence leads to significant variability in residents' baseline proficiency and adds complexity to the teaching process.^12^ In Western China, this variability is critically exacerbated by regional disparities. Many trainees remaining in grassroots hospitals lack prior exposure to basic microneurosurgical concepts due to historical resource limitations. Therefore, our training framework intentionally adopts a ‘back‐to‐basics’ approach, prioritizing the fundamental translation of 2D imaging into 3D spatial cognition before advancing to complex surgical techniques. Consequently, the need for innovative pedagogical approaches has become increasingly urgent. This study evaluated a training model integrating 3D Slicer and 3D printing technology to address these gaps. Its findings, along with insights into the limitations of traditional training and the clinical translation of the intervention, are discussed below with strict adherence to the content of the specified document and referenced literature.

### Training context and limitations of traditional models

Traditional neurosurgery training has long struggled to bridge the divide between theoretical knowledge and clinical practice, particularly in the realms of anatomical cognition and imaging interpretation. Specimen‐based anatomical training, once regarded as the gold standard for anatomical skill acquisition, is plagued by insurmountable constraints: limited availability of cadavers, the requirement for specialized anatomical laboratory equipment (which hinders widespread implementation), the inability to reuse specimens, and poor alignment with real‐world clinical cases.^13^ As a result, residents often rely on textbooks, anatomical atlases, or online videos to learn anatomy. This passive learning approach fails to provide intuitive exposure to real anatomical structures or hands‐on practice opportunities, creating a disconnect between theoretical understanding and clinical application—a gap consistently highlighted in neurosurgical education research.

Another critical bottleneck lies in clinical imaging interpretation, a major challenge for medical trainees. Translating two‐dimensional (2D) imaging data, such as computed tomography angiography (CTA) or magnetic resonance angiography (MRA) scans, into three‐dimensional (3D) spatial relationships remains an obstacle for residents—especially senior trainees.^14^, ^15^ Although many residents have developed the ability to manage wards independently and face pressure to practice independently after graduation, they often lack systematic microneuroanatomical training. This deficiency leads to insufficient understanding of the 3D relationships between key blood vessels, nerves, and lesions during surgical procedures. This limitation is particularly pronounced in minimally invasive neurosurgery, where the steep learning curve and 2D visualization constraints increase the risk of complications such as dural tears or nerve injury. These challenges collectively underscore the urgent need for a training model that can overcome the barrier of converting 2D images to 3D spatial cognition and integrate closely with clinical practice.

### Core value of 3D‐integrated technology: From cognitive breakthrough to active learning

The training model combining 3D Slicer and 3D printing addressed the shortcomings of traditional training through two key mechanisms: overcoming spatial cognition barriers and fostering active learning—aligning with the core conclusions of multiple innovative education studies.

#### 3D‐Slicer: Bridging the 2D‐to‐3D gap in anatomical cognition

The interactive reconstruction capability of 3D Slicer proved pivotal in transforming medical residents' anatomical understanding. By enabling trainees to manually reconstruct intracranial lesions (such as meningiomas and aneurysms) using 3D Slicer, the trainees integrated abstract 2D textbook knowledge and imaging data into visualized 3D structures. This transformation echoes findings from studies on multimodal 3D visualization. These studies confirm that combining digital models with physical learning tools significantly improves novices' interpretation of cross‐sectional anatomy. Additionally, it reduces the difficulty of imaging analysis.

Quantitative assessments in this study showed that scores for spatial anatomical recognition, which focused on identifying predefined anatomical landmarks in thin‐slice imaging, increased significantly after training. This aligns with research demonstrating that 3D volumetric visualization enhances the accuracy and speed of anatomical structure identification compared to 2D methods, particularly for complex subcortical structures like the hippocampus and amygdala. Subjective feedback further confirmed that enhanced understanding of 3D intracranial anatomy and improved ability to conceptualize 3D structures from 2D images were among the most highly rated outcomes. These findings are consistent with evidence that 3D stereoscopic models benefit even trainees with low inherent spatial ability, narrowing performance gaps with high‐spatial‐ability peers.

Notably, this approach also mitigated the impact of variability in residents' baseline proficiency in anatomical knowledge and spatial skills. 3D Slicer provided a standardized hands‐on platform for anatomical reconstruction, ensuring that all trainees—regardless of their initial skill level—gained consistent exposure to 3D anatomical relationships, thereby reducing disparities in spatial cognition.

#### Model construction‐driven problem generation: Stimulating active learning

Traditional training often leaves residents in a passive state, in which they cannot identify problems or raise questions due to limited hands‐on experience and in‐depth understanding. In contrast, the design of this study—centered on “model construction driving problem generation”—transformed learning from passive absorption to active exploration, representing a key innovation emphasized in recent neurosurgical education research. As residents completed the entire process from data segmentation to 3D anatomical model reconstruction, they naturally encountered clinical questions. For example, they asked, “Why is this aneurysm easier to clip from this angle?” or “How can this surgical approach avoid functional brain regions?”

This shift from passive to active learning was directly reflected in the subjective survey: 68% of respondents reported a “significantly improved” ability in “proactive inquiry and problem‐solving,” with an additional 32% reporting “moderately improved.” This outcome aligns with conclusions from simulation training studies, which emphasize that hands‐on, model‐based learning fosters higher order thinking by requiring trainees to engage with realistic clinical dilemmas—an advantage not offered by traditional didactic teaching.

#### Clinical translation and practical outcomes: Closing the “theory‐planning‐practice” loop

The ultimate goal of residency training is to prepare residents for independent clinical practice, and the 3D‐integrated model excelled in translating training gains into tangible clinical capabilities—consistent with the core value of 3D printing in neurosurgery as identified in systematic reviews (Figure 4).

**FIGURE 4:**
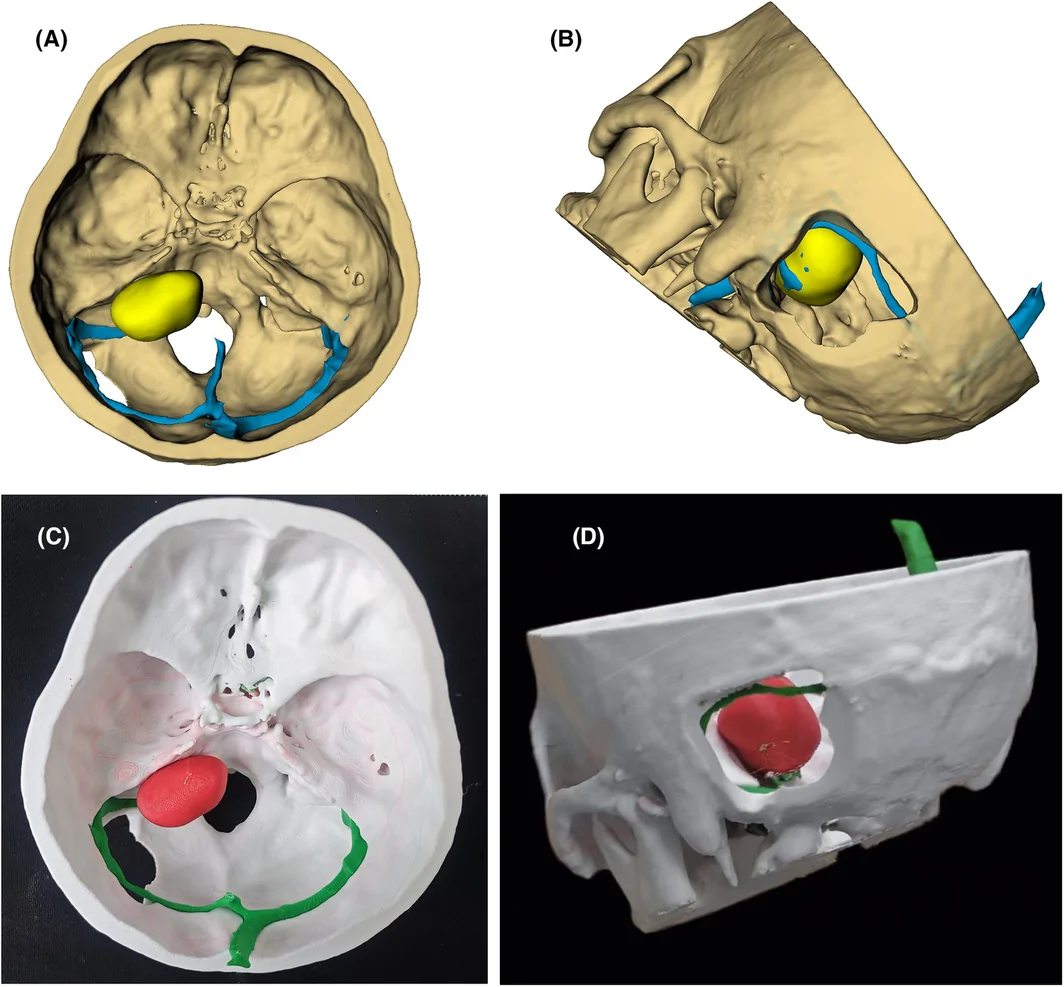
Translating digital planning to physical simulation: A retrosigmoid approach for vestibular schwannoma. (A) 3D‐Slicer digital anatomical reconstruction of the posterior fossa, clearly illustrating the vestibular schwannoma (tumor mass) in relation to the skull base and the critical venous sinus network (including the transverse, sigmoid, and portions of the superior sagittal sinuses). (B) 3D Slicer simulated surgical perspective demonstrating the planned retrosigmoid craniotomy window and the spatial trajectory toward the lesion. (C) The corresponding 1:1 scale 3D‐printed physical phantom, providing residents with a tangible representation of the complex intracranial spatial anatomy. (D) The 3D‐printed surgical model with the retrosigmoid bone window removed. This allows trainees to physically interact with the simulated surgical corridor, actively bridging the gap between theoretical planning and practical spatial orientation.

First, 3D printing technology converted intangible digital models into tangible physical phantoms, creating a closed loop of “theory → planning → practice.” This aligns with findings from the development and validation of neurosurgical phantoms, which demonstrate that 3D‐printed models with realistic anatomical and mechanical properties effectively enhance surgical skill acquisition. Residents who mastered routine case reconstruction using 3D Slicer successfully performed intracerebral hematoma puncture independently using 3D‐printed surgical guides under the supervision of senior physicians—mirroring results from studies where 3D‐printed simulators improved accuracy in procedures like external ventricular drain placement and endoscopic tumor resection. For example, training with 3D‐printed models has been shown to increase optimal EVD placement rates from 55.3% to 84.0% and reduce targeting errors, paralleling the improved precision observed in this study's clinical applications.^16^ This paradigm of utilizing accessible digital models closely mirrors successful educational interventions in other complex surgical fields, notably in Otorhinolaryngology (ENT) surgery. Extensive assessments of educational programs have validated the use of digital models for simulating temporal bone drilling.^17^, ^18^ Just as ENT has effectively utilized these cost‐effective digital frameworks to safely navigate complex bony anatomy, our findings demonstrate that neurosurgery can similarly adopt this model to lower the training threshold for skull base and vascular navigation.

Second, the model supported residents' academic and professional development: some residents applied 3D‐Slicer reconstruction and 3D printing technology to their master's thesis research and successfully completed their thesis defenses. More importantly, by the time of manuscript preparation, all participating residents had transitioned to new work environments and introduced 3D‐printed guide technology to their new positions—reflecting the scalability and transferability of 3D‐based training outcomes, a key conclusion from cost‐effective model evaluations.^13^ This real‐world adoption echoes evidence that low‐cost 3D‐printed models provide viable, scalable options for training programs, even in resource‐limited settings.

### Study limitations

Despite the positive outcomes of this study, several limitations should be acknowledged—many of which are common in neurosurgical simulation research and highlighted in recent reviews.

First, the sample size was small. While the cohort was selected for their consistent clinical background and training duration, this specificity limits generalizability to residents at different training stages or with diverse educational backgrounds—a challenge noted in studies of both 3D‐printed models and virtual reality simulators.^19^ Another limitation is the demographic composition of our cohort, as all participants were male. The demanding nature of neurosurgery in underdeveloped regions of Western China, characterized by heavy emergency workloads and structural resource constraints, currently skews the demographic heavily toward male practitioners.^20^, ^21^ While this reflects the local reality and was beyond the authors' control, it is not an aspiration globally. We recognize that actively encouraging the inclusion of women and nonbinary individuals is vital for the specialty's future, and we hypothesize that developing accessible, technology‐driven training models like ours could help reduce barriers and attract a more diverse demographic to neurosurgical residency.

Second, the self‐controlled before‐ and after‐design, while reducing interindividual variability, cannot fully exclude the influence of “natural growth” through routine clinical work. This limitation mirrors concerns in simulation training research, where distinguishing intervention effects from concurrent clinical experience remains a methodological challenge. A parallel control group receiving traditional training would help isolate the 3D model's specific impact, as recommended in systematic reviews of neurosurgical education interventions.

Third, the study lacked long‐term follow‐up data. While immediate skill improvements were observed, research on simulation training indicates that skill retention and clinical translation require assessment over extended periods (6–24 months) to confirm sustained benefits—particularly for complex procedures like aneurysm clipping or endoscopic resection. Without such data, it is impossible to verify whether the observed gains persist in independent clinical practice.

Finally, the assessment of surgical skill relied partly on subjective feedback, which may be prone to social desirability bias. This aligns with broader calls in the field for standardized, objective assessment tools to evaluate simulation‐based training outcomes, such as motion analysis or error counting metrics.

## CONCLUSION

In the context of microneurosurgery, the training model integrating 3D Slicer and 3D printing technology effectively addresses the core challenges of standardized resident training—consistent with the transformative role of 3D technology in neurosurgery identified in comprehensive reviews. It overcomes the limitations of traditional specimen‐based anatomical training and 2D imaging interpretation, bridges the 2D‐to‐3D gap in spatial cognition (as validated in multimodal visualization studies),^15^ stimulates active learning (a key innovation in modern medical education), and achieves a closed loop of “theory → planning → practice” (critical for skill translation).

Tangible outcomes—including improved scores in spatial anatomical recognition, enhanced proactive problem‐solving abilities, successful clinical application of 3D‐printed guides, and technology transfer to new workplaces—validate the model's value in preparing residents for independent practice. These results align with cumulative evidence that 3D printing and digital visualization technologies improve training efficiency, procedural accuracy, and trainee confidence across neurosurgical subspecialties.

While small sample size, self‐controlled design, and limited long‐term follow‐up restrict generalizability—challenges common in the field—the model provides a scalable, clinically relevant framework for neurosurgery training. Future research expanding to diverse trainee cohorts, incorporating control groups, and conducting long‐term follow‐up will further validate its efficacy—advancing the standardization of micro‐neurosurgical training and ultimately improving patient care, as advocated in recent neurosurgical education guidelines.

## AUTHOR CONTRIBUTIONS

**Li Xueyu:** Conceptualization; methodology; software; data curation; investigation; resources; writing – review and editing; funding acquisition; validation; project administration. **Fan Yuanxian:** Formal analysis; visualization; writing – original draft; writing – review and editing. **Chen Jiaxing:** Investigation; software; validation. **Li Chuanyu:** Investigation; validation; software. **Chen Yuanbiao:** Software; investigation; validation (specifically involved in 3D‐Slicer software processing, experimental execution, and validation of imaging reconstructions). **Huang Haineng:** Software; investigation; validation. **Huang Huadong:** Data curation; methodology; conceptualization; resources. **Luo Kunxiang:** Investigation; validation. **Wei Shiyin:** Investigation; validation. **Tan Rong:** Investigation; validation.

## FUNDING INFORMATION

This work was supported by the Self‐funded Scientific Research Project of the Health Commission of Guangxi Zhuang Autonomous Region (Grant No. Z‐L20220875).

## Data Availability

The data that support the findings of this study are available from the corresponding author upon reasonable request.
